# Multi-region ultrasound-based deep learning for post-neoadjuvant therapy axillary decision support in breast cancer

**DOI:** 10.1016/j.ebiom.2025.105916

**Published:** 2025-09-10

**Authors:** Ziyin Li, Ning Mao

**Affiliations:** a Department of Radiology, Yantai Yuhuangding Hospital, Qingdao University, Yantai, Shandong, 264000, PR China; b Big Data and Artificial Intelligence Laboratory, Yantai Yuhuangding Hospital, Qingdao University, Yantai, Shandong, 264000, PR China

Breast cancer is one of the most common malignancies in women, and axillary lymph node (ALN) status critically guides prognosis and treatment. Sentinel lymph node biopsy (SLNB), the preferred method to assess axillary response after neoadjuvant therapy (NAT), often has a false-negative rate (FNR) above 10%, leading to unnecessary axillary lymph node dissection (ALND) and associated complications.^1^ Recent years, the central challenge has been how to reliably identify patients with axillary pathological complete response (pCR) who may avoid ALND, a challenge that traditional clinicopathological and imaging prediction methods have not adequately solved.^2^^,^^3^ In this context, the rapid progress of artificial intelligence (AI) offers a chance to rethink axillary management and develop new frameworks for supporting clinical decision-making.

In the recent issue of *eBioMedicine*, Liu and colleagues^4^ proposed iShape, a deep learning framework designed to predict ALN pCR by leveraging longitudinal, multi-region ultrasound imaging data. Beyond its excellent performance, with AUCs from 0.950 to 0.971 across three external validation datasets and a reduction of SLNB's FNR from 13.4% to 3.6%, the clinical significance of iShape lies in its potential to refine axillary management. By identifying patients likely to achieve ALN pCR, iShape could help spare patients from unnecessary ALND and its long-term complications, thus moving surgical decision-making toward a more individualised, risk-adapted approach.

Metastatic ALNs originate from the primary tumour, they share certain phenotypic traits yet also exhibit region-specific differences. Previous studies typically analysed only tumours or ALN regions, neglecting this complementarity and limiting predictive accuracy.^5^^,^^6^ Moreover, NAT is a dynamic process, yet most approaches did not use longitudinal imaging to capture treatment-induced changes. A key strength of this study is the use of a shared–private framework, enabling iShape to learn both shared and region-specific representations from longitudinal ultrasound images of the primary tumour (pre- and post-NAT) and ALN (pre-NAT). This design may provide a new paradigm for processing longitudinal, multi-region imaging data in oncology.

Equally important is interpretability, a prerequisite for clinical trust in AI-based decision support. Liu and colleagues^4^ employed Grad-CAM to visualise the regions that iShape prioritised during prediction. Notably, the model focused on the lymph-node cortex and tumour centre in pCR cases, whereas attention was directed to peri-tumoral and peri-cortical margins in non-pCR cases. These patterns closely align with established pathological knowledge, suggesting that iShape capture meaningful information.^7^^,^^8^ Moreover, RNA sequencing analysis offered molecular-level validation: patients predicted as non-pCR by iShape exhibited enrichment in immune checkpoint, metabolic, and tumour microenvironment pathways.

Despite the promising results, several open questions remain. First, the study was retrospective and conducted in a Chinese population; validation in prospective, multi-ethnic, and multi-centre trials will be essential for generalisation. Second, current framework relies on static ultrasound images, whereas dynamic ultrasound video may provide richer temporal information on tumour and lymph node changes during therapy, and could potentially improve predictive accuracy. Third, while iShape reduced SLNB's FNR when used as an adjunct, its performance as a standalone decision tool for omitting ALND needs further evaluation, particularly in cases where fewer than three sentinel nodes are retrieved.^9^ Finally, missing clinical or imaging data, inevitable in real-world practice, must be addressed if AI tools are to be embedded into clinical workflows.

Looking ahead, the rapid advancement of foundation models offers new opportunities for breast cancer imaging. Future frameworks may leverage pre-trained foundation models that integrate diverse data types, including imaging, clinical, molecular, and other relevant information, enhancing robustness and cross-institutional generalisability. Federated learning approaches can further expand these capabilities, enabling multiple institutions to collaboratively train AI models without sharing sensitive raw data, thus facilitating large-scale, privacy-preserving development. Moreover, incorporating reasoning techniques such as Chain-of-Thought, which enables stepwise, transparent decision-making, could further improve both diagnostic performance and interpretability in clinical practice.

## Contributors

Both authors contributed to conceptualisation, writing, reviewing, editing and have read and approved the published version of the manuscript.

## Declaration of interests

The author has no conflicts of interest to disclose.
